# Transcriptome data from silica-preserved leaf tissue reveal gene flow patterns in a Caribbean bromeliad

**DOI:** 10.1093/aob/mcae002

**Published:** 2024-01-05

**Authors:** Natalia Ruiz-Vargas, Karolis Ramanauskas, Alexa S Tyszka, Eric C Bretz, May T S Yeo, Roberta J Mason-Gamer, Joseph F Walker

**Affiliations:** Department of Biological Sciences, the University of Illinois at Chicago, Chicago, IL 60607, USA; Department of Biological Sciences, the University of Illinois at Chicago, Chicago, IL 60607, USA; Department of Biological Sciences, the University of Illinois at Chicago, Chicago, IL 60607, USA; Department of Biological Sciences, the University of Illinois at Chicago, Chicago, IL 60607, USA; The Sainsbury Laboratory, University of Cambridge, Bateman Street, Cambridge CB2 1LR, UK; Department of Genetics, Downing Site, University of Cambridge, Cambridge CB2 3EJ, UK; Department of Biological Sciences, the University of Illinois at Chicago, Chicago, IL 60607, USA; Department of Biological Sciences, the University of Illinois at Chicago, Chicago, IL 60607, USA

**Keywords:** Transcriptomics, historical RNA, Bromeliaceae, *Pitcairnia*, Dominican Republic, Hispaniola, phylotranscriptomics, RNA extraction, population genetics, *Pitcairnia*, Bromeliaceae, Extracción de ARN, Filogenética, Genética de Poblaciones, Plasticidad Fenotípica, República Dominicana, Ayiti, Hispaniola

## Abstract

**Background and Aims:**

Transcriptome sequencing is a cost-effective approach that allows researchers to study a broad range of questions. However, to preserve RNA for transcriptome sequencing, tissue is often kept in special conditions, such as immediate ultracold freezing. Here, we demonstrate that RNA can be obtained from 6-month-old, field-collected samples stored in silica gel at room temperature. Using these transcriptomes, we explore the evolutionary relationships of the genus *Pitcairnia* (Bromeliaceae) in the Dominican Republic and infer barriers to gene flow.

**Methods:**

We extracted RNA from silica-dried leaf tissue from 19 *Pitcairnia* individuals collected across the Dominican Republic. We used a series of macro- and micro-evolutionary approaches to examine the relationships and patterns of gene flow among individuals.

**Key Results:**

We produced high-quality transcriptomes from silica-dried material and demonstrated that evolutionary relationships on the island match geography more closely than species delimitation methods. A population genetic examination indicates that a combination of ecological and geographical features presents barriers to gene flow in *Pitcairnia*.

**Conclusions:**

Transcriptomes can be obtained from silica-preserved tissue. The genetic diversity among *Pitcairnia* populations does not warrant classification as separate species, but the Dominican Republic contains several barriers to gene flow, notably the Cordillera Central mountain range.

## Abstract (es)

**Premisa del Estudio:**

La sequenciación de transcriptomas es una técnica costo-efective que permite estudiar múltiples preguntas. Sin embargo, para secuenciar transcriptomas, el tejido suele mantenerse bajo condiciones especiales, como el ultra-congelamiento inmediato. Aquí demostramos que el ARN puede ser obtenido de muestras preservadas en silica a temperatura ambiente. Usando estos transcriptomas exploramos las relaciones evolutivas de *Pitcairnia* (Bromeliaceae) en la República Dominicana e inferimos barreras para el flujo genético.

**Métodos:**

Extraímos ARN de tejido preservado en sílica de 19 individuos de *Pitcairnia* colectados en la República Dominicana. Utilizamos técnicas macro y microevolutivas para examinar las relaciones y patrones de flujo genético entre los individuos.

**Resultados Clave:**

Produjimos transcriptomas de alta calidad a partir de material preservado en sílica y demostramos que las relaciones evolutivas en la isla coinciden más con la geografía que con la delimitación de especies actual. Los análisis de genética de poblaciones indican que factores ecológicos y geográficos son barrera para el flujo genético.

**Conclusiones:**

Transcriptomas de alta calidad pueden ser obtenidos de tejido preservado en sílica. La diversidad genética entre las poblaciones de *Pitcairnia* no amerita una clasificación en especies separadas, pero la República Dominicana contiene varias barreras para el flujo genético, notablemente, La Cordillera Central.

## INTRODUCTION

Transcriptome sequence data are easily transferable between studies and allow researchers to pursue a broad range of evolutionary questions, including the inference of palaeopolyploidy events (Jiao *et al.*, 2011), phylogenomic analyses (Wickett *et al.*, 2014; Yang *et al.*, 2017), uncovering putatively adaptive gene duplications (Brockington *et al.*, 2015) and estimating population structure (Raduski *et al.*, 2021). Transcriptome sequencing is more affordable than whole-genome sequencing yet provides data that can be used to answer many similar questions. Unlike affordable sequencing alternatives, such as target enrichment, it does not require predesigned bait datasets (Dodsworth *et al.*, 2019; Johnson *et al.*, 2019), and it allows for more precise identification of coding regions in comparison to genome skimming (Zeng *et al.*, 2018) and provides protein-coding sequences, unlike restriction site-associated sequencing (Andrews *et al.*, 2016; Eaton *et al.*, 2017).

Although most sequencing approaches for evolutionary studies of non-model organisms use DNA, transcriptome sequencing requires RNA, which is less stable and degrades faster. Therefore, general guidelines have advised only the use of samples preserved in solutions such as RNA-later™ or flash-frozen in liquid nitrogen and maintained at temperatures of −70 °C or lower (Logeman *et al.*, 1987; Vennapusa *et al.*, 2020). These requirements for sample preparation have greatly limited the scope of transcriptomic approaches, especially for taxa from regions with limited cryo-preservation resources.

The recent emergence of the historical RNA field has brought forth the possibility that intact RNA might be obtained from unpreserved tissue. In metazoans, transcriptomic sequences have been obtained from a 130-year-old Tasmanian tiger (Mármol-Sánchez *et al.*, 2023). RNA allows for superior genome annotation, identification of microRNAs and analyses of transcriptomic profiles, making it invaluable for understanding genome evolution. In the plant world, most RNA from unpreserved samples has been obtained from seeds, such as ancient maize kernels (Fordyce *et al.*, 2013). Seeds require RNA for germination. In cases of extreme seed longevity, such as the date palm seeds germinated after 2000 years (Sallon *et al.*, 2008), the RNA must have been intact. It is unknown how long plant RNA might survive without protective seed-specific mechanisms. One key question in the botanical world remains whether leaf and other tissues collected in the field might provide transcriptomes even when not preserved.

A recent study by He *et al.* (2022) found comparable quality between transcriptomes obtained from plant tissue dried in silica gel, then preserved at −20 °C and those from samples flash-frozen in liquid nitrogen. These results suggest that previously imposed limitations of tissue preservation might be overly cautious, and the study provided a framework for obtaining transcriptomes from a broader range of samples. To explore this possibility further, we investigated whether acceptable transcriptomes could be obtained from field-collected plant tissue preserved in silica gel at room temperature for ≤6 months. These conditions replicate those of even the most prolonged fieldwork trips and test the feasibility of obtaining transcriptome data from any species in the world where collections are possible.

The focal group of this study are the flowering plants in the genus *Pitcairnia*, specifically those from the Dominican Republic. *Pitcairnia* is the second largest genus in the Neotropical flowering plant family Bromeliaceae, with >300 species (Luther, 2014). A molecular phylogenetic analysis based on four chloroplast markers suggested that all Caribbean species of the genus are monophyletic (Schubert, 2017). Unlike other islands in the Caribbean, where each island contains a single species of *Pitcairnia*, Ayiti (Hispaniola; note that Ayiti is the name given to the island by the Taino people, the indigenous group present at the time of colonization, and the spelling differentiates it from the country of Haiti, located on the island) contains up to six named species (Acevedo-Rodríguez and Strong, 2012), inferred from the high level of morphological disparity observed among individuals. *Pitcairnia* grows throughout the diverse landscape and varied niches of the Dominican Republic, which might have promoted speciation and led to the diversification and increased morphological disparity of *Pitcairnia*.

In this study, we use transcriptome data to examine the diversity of *Pitcairnia* native to the Dominican Republic. We demonstrate that transcriptome data for evolutionary studies can be obtained from field-collected silica-dried tissue. In examinations of molecular diversity, we find that the morphology-based species delimitation in *Pitcairnia* of the Dominican Republic is not supported. We do uncover barriers to gene flow that correspond to the topography and ecological characteristics of the Dominican Republic, but they are not sufficient to lead to speciation. This study provides a framework for researchers seeking to obtain and analyse transcriptome data from field-collected samples.

## MATERIALS AND METHODS

### Data availability

Raw paired-end sequence reads were deposited in the NCBI Sequence Read Archive (SRA), project identity PRJNA982071. Analysis scripts can be found at: https://github.com/karolisr/pitcairnia-dr-nrv. Assemblies, alignments and trees are available at: https://zenodo.org/record/8021855.

### Tissue collection, RNA extraction and sequencing

**Fig. 1.**
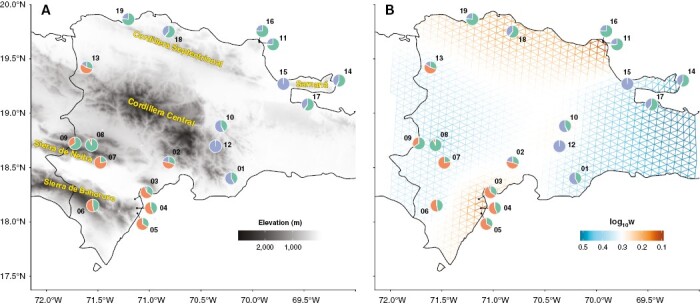
Maps of the Dominican Republic highlighting collection sites of *Pitcairnia* specimens. Pie charts are labelled with sample numbers and show the proportion of each layer inferred to have contributed to the ancestry of each sample based on the conStruct analysis. (A) Darker areas correspond to higher elevations, with major mountain ranges labelled. (B) The fast estimation of effective migration surfaces (FEEMS) analysis. Warmer colours correspond to areas inferred to have less gene flow.

Leaf tissue was collected from 19 *Pitcairnia* individuals from across the distribution of the genus in the Dominican Republic (Fig. 1). Samples were collected in 2021 from 28 June to 2 July or from 11 to 14 September, with the corresponding permits from the Ministry of Environment of the Dominican Republic. Samples were placed immediately in resealable plastic bags filled with silica gel. After the collection trips, samples were stored at room temperature before RNA extraction.

Either 3 or 6 months after collection, ~40 mg of tissue from each sample was ground to a fine powder in 1.5 mL tubes mounted in a liquid nitrogen-cooled mini mortar (model no. H37260-0100; Bel-Art SP Scienceware, Wayne, NJ, USA). RNA was extracted from the ground leaf tissue using the Sigma-Aldrich Spectrum™ Plant Total RNA Kit with a DNAse treatment, and the extracted RNA was stored at −80 °C. Minor modifications made to the protocol are outlined in the Supplementary Data (Fig. S1). The University of Illinois at Chicago (UIC) Sequencing Core assessed the RNA quality using the RNA ScreenTape. Library preparation was performed using unstranded paired-end total RNAseq with rRNA depletion. Paired-end 150 bp sequencing was carried out on a single lane of an Illumina NovaSeq 6000 System, flow cell type S4, also at the UIC Sequencing Core.

Raw sequence reads for two outgroup species, *Pitcairnia albiflos* (SRR2518104) and *Pitcairnia staminae* (SRR2518089), were obtained from the NCBI SRA database using the fastq-dump utility from the SRA-Toolkit package v.3.0.0 (https://hpc.nih.gov/apps/sratoolkit.html).

### Morphological data

Specimens for the field-collected samples were pressed and deposited in the National Herbarium of the Dominican Republic (JBSD) (Supplementary Data Table S1). Trichome density, leaf width and length, and marginal spine size were measured because they were the most variable among collection sites. Furthermore, trichome density is considered important for specimen identification (Teodoro Clase, Botanical Garden of Santo Domingo, pers. comm., December 2022). Trichome density was estimated in the field and classified into three categories: high, medium and low. Leaf width, length and marginal spine size were measured from the dried specimens. The average measurements of the three largest leaves are reported.

Two approaches were used to classify the samples. The first approach was to have a taxonomist specialized in Dominican flora assign species names to the collections. The second was to use the species names given to the populations based on geolocation data from herbarium specimens. Geolocation-based assignment was unable to classify Samples 13 and 19 because other herbarium specimens from their location were identified only to genus (i.e. *Pitcairnia* sp.). Sample 13 also could not be classified by the taxonomist and consequently remained undetermined as *Pitcairnia* sp. The geolocation-based classification identified four samples of *Pitcairnia elizabethae*, four *Pitcairnia fuertesii*, three *Pitcairnia domingensis*, two *Pitcairnia ariza-juliae*, two *Pitcairnia samuelsonii* and two *P. jimenezii*. In contrast, the taxonomist classification identified eight *P. elizabethae*, seven *P. domingensis* and three *P. fuertesii*.

### Read processing and transcriptome assembly

Random sequencing errors in the raw reads were identified and corrected using Rcorrector v.1.0.4 (Song and Florea, 2015). Uncorrectable reads were removed using the unfixable_filter.py script from Morales-Briones *et al.* (2021). Trimmomatic v.0.39 (Bolger *et al.*, 2014) was used to trim and remove low-quality sequences with the settings ‘SLIDINGWINDOW:4:5 LEADING:5 TRAILING:5 MINLEN:25’. Kraken2 v.2.1.2 (Wood *et al.*, 2019) was used to filter human, bacterial, protozoan and fungal reads using the Kraken2 PlusPF database (https://genome-idx.s3.amazonaws.com/kraken/k2_pluspf_20220607.tar.gz) with default settings. Kraken2, with a confidence value of 0.1, was also used to remove plastid and mitochondrial reads using their respective pre-made databases from Kakapo (Ramanauskas and Igic, 2023). All reads not identified as contamination or organelle genomes were retained as nuclear reads.

For each sample, the nuclear reads were assembled using Trinity v.2.11 (Haas *et al.*, 2013), with default settings and the option ‘--no_normalize_reads’. Chimeric sequences were removed following the procedure of Yang and Smith (2013), using Blastx v.2.13.0 (Altschul *et al.*, 1990), with a protein database composed of the *Arabidopsis thaliana* (TAIR 10), *Oryza sativa* (IRGSP 1.0), *Zea mays* (B73 REFERENCE-NAM-5.0) and *Ananas comosus* (F153) coding sequences (hereafter, AOZA database), downloaded from PlantEnsembl (Bolser *et al.*, 2016). The program Corset v.1.09 (Davidson and Oshlack, 2014), with mapping conducted using Salmon v.0.9.1 (Patro *et al.*, 2017), was used to filter for the best transcript. The open reading frames were predicted using Transdecoder v.5.5.0 (https://github.com/TransDecoder/TransDecoder), and predictions were guided by the AOZA database. To remove further possible contamination, only genes with 70 % nucleotide sequence similarity, as inferred by Blastn v.2.13.0, compared with the corresponding nucleotide sequences in the AOZA database, were retained.

The same procedure was conducted for both the plastid and the mitochondrial datasets to infer organelle transcriptomes, with a database composed of (NC_026220.1: *Ananas comosus*; NC_000932.1: *Arabidopsis thaliana*; NC_031333.1: *Oryza sativa*; and NC_001666.2: *Zea mays*) for the plastid and (NC_007982.1: *Zea mays*; NC_007886.1: *Oryza sativa*; and NC_037304.1: *Arabidopsis thaliana*) for the mitochondrion. For each predicted organelle transcriptome, the longest single transcript corresponding to an organelle protein-coding sequence was retained for downstream analysis.

### Organelle phylogenetic analyses

The nucleotide sequences for individual plastid protein-coding sequences were aligned using Prank v.170427 (Loytynoja and Goldman, 2008), with default settings. Alignments were cleaned for a minimum column occupancy of 10 % using the Phyx v.1.2 (Brown *et al.*, 2017) program *pxclsq*. All genes were then concatenated using the Phyx program *pxcat*, and the plastid tree was inferred using maximum likelihood as implemented in IQ-TREE v.1.6.12 (Nguyen *et al.*, 2015), with default model selection, the proportional partition model (Chernomor *et al.*, 2016) and 1000 UFBoot2 (Hoang *et al.*, 2018) replicates. The same procedure was performed for the mitochondrial protein-coding sequences.

### Nuclear phylogenetic analyses

Cd-hit v.4.8.1 (Fu *et al.*, 2012), with the settings ‘-c 0.99 -n 10 -r 0’, was run on each nuclear transcriptome to reduce redundancy. All-by-all Blastn was used to identify sequence similarity of combined transcriptomes, setting the e-value to 10 and max-target-seqs to 1000. The clusters of similar sequences were refined using mcl v.14-137 (Van Dongen, 2000), with an inflation value of 1.4. Only clusters with at least four taxa, hereafter referred to as homologue clusters, were retained.

Mafft v.7.490 (Katoh and Standley, 2013), with the settings ‘--auto --maxiterate 1000’, was used to align the homologue clusters individually. The resulting alignments were cleaned with *pxclsq* to ensure a minimum column occupancy of 10 %. Homologue trees were inferred using IQ-TREE, with 1000 UFBoot2 replicates and automated model selection. Long branches with relative values of ≥0.02 substitutions per site (subs/site) that were ten times longer than the sister branch and/or that had an absolute value of ≥0.03 or more were removed from the homologue tree. After each round, the number of branches removed for each tip by this procedure was used to estimate the removal rate for each individual (number of sequences from an individual removed/number of sequences for the individual before the removal procedure). Any homologue tree with a branch >0.2 subs/site and at least four taxa on both sides of the branch was split on the branch and treated as separate homologues for downstream analysis. The same process with the same settings was repeated a second time.

Orthology was inferred using the maximum inclusion method (Yang and Smith, 2014), setting the relative value to 0.02 subs/site, the absolute value to 0.03 subs/site, and requiring a minimum of ten taxa for each orthologue. Orthologue alignments were extracted from the alignments used to infer homologue trees in the same manner as Wheeler *et al.* (2022). Individual gene trees were inferred using IQ-TREE, with default model selection and 1000 UFBoot2 replicates. The individual relationships were estimated using the maximum quartet support species tree method as implemented in Astral v.5.7.8 (Zhang *et al.*, 2018). Support values for the Astral tree were inferred using the coalescent-based quartet frequency method (Sayyari and Mirarab, 2016). Molecular branch lengths were applied to the Astral tree using the quadripartition concordance approach as implemented in Bes (Walker *et al.*, 2022).

### Transcriptome quality control analyses

Transrate v.1.0.3 (Smith-Unna *et al.*, 2016), with default settings, was used to assess the quality of each transcriptome assembly. Transrate was run with the dependencies SNAP v.1.0.0 (Zaharia *et al.*, 2011) and Salmon v.0.6.0 (Patro *et al.*, 2017). The alignment edit distance, *s*(*C*_nuc_) value, was calculated for every contig and summarized by finding the average *s*(*C*_nuc_) value across all contigs of the transcriptome. The *s*(*C*_nuc_) value could not be calculated for the mitochondrion of either outgroup, because they had insufficient reads for the analysis. BUSCO v.5.5.0, with the dependencies hmmsearch v.3.1 (Potter *et al.*, 2018) and metaeuk v.6.a5d39d9 (Levy Karin *et al.*, 2020), and using the Liliopsida database and default settings, was used to infer the completeness of each transcriptome.

### Variant-calling workflow

The outgroup taxa (*P. albiflos* and *P. staminae*) were not included in the variant-calling procedure. A reference was selected for each orthologue by identifying the longest sequence from among the ingroup taxa. STAR v.2.7.10a (Dobin *et al.*, 2013) was used to map the processed sequence reads from each sample to the reference, with the setting ‘--twopassMode’. The Picard command line tools (Picard Toolkit, 2019) were used to filter potential PCR duplicate reads from each alignment. The genotypes, including single nucleotide polymorphisms (SNPs) and invariant sites, were identified using FreeBayes v.1.3.6, with all samples assigned to a single population (Garrison and Marth, 2012). The genotype calls were filtered by removing sites that met any of the following criteria: (1) the site was inferred to have more than two alleles segregating across all samples; (2) the site was called polymorphic but had a ‘Phred-scaled quality score’ (QUAL field in the VCF file; 10log10prob[no variant]) <20; (3) the read depth of sites was lower than ten in any of the 19 samples; (4) the site had one allele overrepresented in heterozygous samples (allele balance; AB < 0.25 or AB > 0.75); (5) the site had AB > 0.05 in homozygous samples; or (6) the site had variants with a frequency <0.05 across all samples (minor allele frequency ≤ 0.05).

### Principal component analysis

The R package SNPRelate v.1.32.1 (Zheng *et al.*, 2012) was used to filter genotypes and perform a principal component analysis (PCA) (McVean, 2009). Linkage disequilibrium (LD) was filtered from the genotypes using the function snpgdsLDpruning, with LD threshold set to zero (i.e. one SNP per locus was used) and the missing sample rate threshold set to one sample (ld.threshold = 0.0, missing.rate = 1/19 + 1e-10). The PCA was conducted using the function snpgdsPCA.

### Genetic structure analysis using conStruct

Genetic structure analysis was performed using R package conStruct v.1.0.5 (Bradburd *et al.*, 2018). Only loci present in ≥15 of the 19 samples were used. Potential linkage disequilibrium bias was reduced by randomly choosing one SNP per locus. Samples were not assigned to populations (i.e. each sample was treated as a separate population). After running cross-validation analysis with four chains, comparing *k*-values 1–6 under spatial and non-spatial models, a spatial model with three layers (*k* = 3) was chosen, and a run with four chains of 20 000 generations was performed. Spatial information for the conStruct analysis was incorporated using the geographical coordinates of the collected samples.

### Fast estimation of effective migration surfaces

A fast estimation of effective migration surfaces (FEEMS) (Marcus *et al.*, 2021) analysis was performed to infer and visualize potential barriers to gene flow. The same set of SNPs was used for this analysis as for the conStruct analysis. The optimal smoothness parameter (*λ* value) was inferred using cross-validation. An outline of the island of Ayiti was created manually using the online *Polyline* tool (https://www.keene.edu/campus/maps/tool). A Geodesic Discrete Global Grid Systems grid intersecting the outline of the island was generated using the R package dggridR (Barnes and Sahr, 2017). Grid size must be selected by a user; therefore, it is somewhat arbitrary. FEEMS assigns samples to the closest node on the grid. If a grid is too large, all samples will be assigned to a single node. In contrast, performance increases non-linearly with increasing node density. When considering these factors, we chose a grid size that assigned samples with a pairwise distance >5 km to a unique node.

## RESULTS

### RNA extraction, quality assessment and transcriptome assembly

Across the 19 samples, the RNA integrity (RIN) values ranged from 2.3 to 7.4 (Table 1; Supplementary Data Table S2). The number of sequenced reads ranged from a minimum of 77 268 317 for Sample 02 to a maximum of 99 484 187 for Sample 01. Slightly less than half of the reads from each sample were filtered as human, protozoan, bacterial or fungal contamination. Of the remaining reads for Sample 01, 46 116 739 reads were inferred to be nuclear, 865 000 were inferred to be plastid, and 1 407 674 were inferred to be mitochondrial. For Sample 02, 46 578 119 reads were inferred to be nuclear, 6 349 726 reads were inferred to be plastid, and 1 682 695 reads were inferred to be mitochondrial. After a final filtering step that required all predicted transcripts to have ≥70 % nucleotide similarity with the coding sequences of *Arabidopsis thaliana*, *Oryza sativa*, *Zea mays* or *Ananas comosus*, we found that Sample 01 had a predicted 23 185 nuclear genes, 37 chloroplast genes and 36 mitochondrial genes, and Sample 02 had 24 990 nuclear genes, 38 chloroplast genes and 39 mitochondrial genes.

**Table 1.** Summary results of the RNA extractions and assembled transcriptomes used in the study. *Pitcairnia albiflos* and *Pitcairnia staminae* were downloaded from the NCBI Sequence Read Archive and therefore do not have total RNA or RNA integrity (RIN) values.

Sample name	Total RNA (ng)	RIN	Total sequence reads	Nuclear sequences	Chloroplast sequences	Mitochondrial sequences
Sample 01	510	2.3	99 484 187	46 578 119	6 349 726	1 682 695
Sample 02	840	4.3	77 268 317	46 116 739	865 000	1 407 674
Sample 03	528	3.8	81 670 840	45 084 923	927 284	1 320 711
Sample 04	4930	5.4	77 788 478	51 905 175	3 207 685	2 675 367
Sample 05	510	3.2	89 263 036	49 957 936	5 133 436	1 909 637
Sample 06	390	4.3	99 040 548	65 979 048	2 596 433	3 218 196
Sample 07	2380	3.7	87 455 923	46 613 054	8 306 422	2 209 659
Sample 08	1581	7.4	95 356 788	56 232 278	99 537	2 066 630
Sample 09	6600	4.8	86 434 374	49 579 100	3 901 460	1 678 802
Sample 10	3100	4.1	80 217 537	49 375 135	3 585 535	2 127 142
Sample 11	1020	5.7	90 185 382	45 720 319	866 775	1 421 561
Sample 12	600	5	86 422 214	60 871 492	2 105 689	1 678 563
Sample 13	1110	5	98 358 395	58 213 213	3 565 910	2 246 538
Sample 14	899	4.9	95 451 297	52 588 180	1 376 271	1 884 013
Sample 15	690	3.7	81 388 060	50 583 456	4 039 181	2 913 524
Sample 16	990	4.1	84 733 604	49 143 522	4 013 399	2 781 999
Sample 17	1488	5.7	85 161 150	45 201 696	478 966	1 656 652
Sample 18	406	4.2	79 558 466	45 963 594	974 380	2 267 451
Sample 19	1290	6.1	88 947 285	52 751 861	1 804 642	1 206 667
*P. albiflos*	–	–	56 976 403	40 661 866	325 139	46 110
*P. staminae*	–	–	51 790 009	43 995 454	57 717	9067

### Quality assessment of assembled transcriptomes

**Fig. 2.**
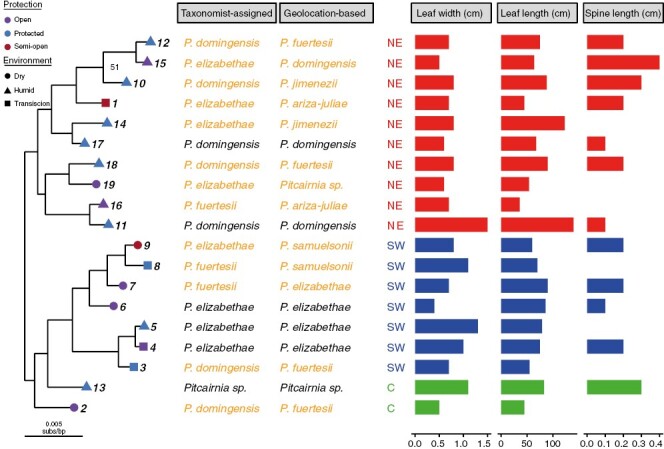
Phylogenetic relationships inferred from 11 683 nuclear gene trees, taxonomic classifications and morphological traits for 19 samples of *Pitcairnia*. The phylogenetic relationships among individuals do not correspond to taxonomic classifications or morphological traits. The tree was inferred with a coalescent-based maximum quartet species tree method, and support for the relationships is given by local posterior probabilities (LPPs). The LPP values for all poorly supported relationships (LPP < 95 %) are labelled. Branch lengths are in units of substitutions per site, and the values depicted are the mean lengths of corresponding branches from concordant gene trees. The shapes and colours on the tips of the tree correspond to the environmental conditions of the sample location, and the number corresponds to the individual sample number. The species names were assigned in two ways: by a taxonomic specialist and based on the geolocation data. Assigned species names are aligned with the corresponding tree tip labels, as are three major morphological characters used to help guide taxonomic identification. The value of each morphological character is an average of three individuals collected at the location. The colours of the bar charts correspond to the region of origin of each sample.

**Fig. 3.**
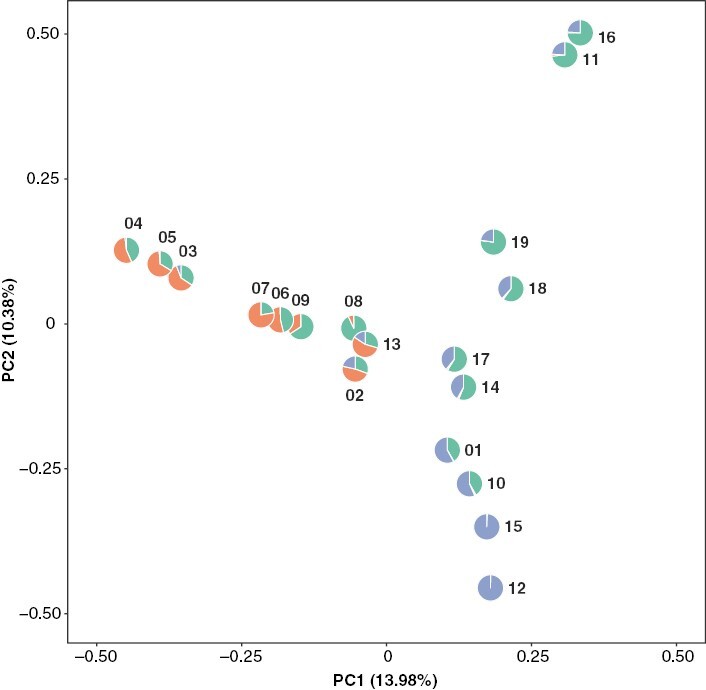
Principal component analysis of *Pitcairnia* individuals. The sample numbers for each individual are shown, and the pie charts correspond to the proportion of each layer inferred to have contributed to the ancestry of the sample based on the conStruct analysis (as in Fig. 1). Samples from the north-east and south-west sides of the Cordillera Central were differentiated by the first principal component (PC1).

The collection date (Table 2) did not have an impact on the clustering of the samples, either phylogenetically (Fig. 2) or by PCA (Fig. 3). The two most genetically similar samples, Samples 11 and 16, were collected 3 months apart. During the long-branch removal process for the homologue trees, we did not see a biased removal of long branches associated with the silica-preserved samples (Table 2). The removal rate ranged between ~6 % for Sample 11 and 12 % for the liquid nitrogen-stored outgroup, *Pitcairnia staminae*. In the second round of tip removal, the removal rate ranged from 0.67 % of tips removed for Sample 17 to 1.6 % of tips removed for the outgroup *P. staminae*. The number of genes with predicted sequence similarity of ≥70 % with the pineapple genome ranged from 4500 for Sample 08 to 27 264 for Sample 07. The percentage missing of the BUSCO Liliopsida universal orthologue set ranged from 97.7 % for Sample 08 to 27.7 % for the outgroup *Pitcairnia albiflos*. The *s*(*C*_nuc_) value for the transcriptomes ranged from 0.98 for Sample 15 to 0.99 for *P. staminae*. The sample value for the chloroplast ranged from 0.98 for Sample 16 to 0.99 for *P. albiflos*; these same values for the mitochondrial dataset ranged from 0.97 for Sample 17 to 0.99 for Sample 06.

**Table 2.** Quality assessments conducted to verify the reliability of the transcriptome data.

Sample name	Age before extraction	Genes with 70 % similarity to *Ananas comosus*	Percentage of BUSCO missing	Removal rate (%) round 1	Removal rate (%) round 2	Average *s*(*C*_nuc_) nuclear sequences	Average *s*(*C*_nuc_) chloroplast sequences	Average *s*(*C*_nuc_) mitochondrial sequences
Sample 01	6 months	24 990	48.1	7.42	0.81	0.98	0.98	0.98
Sample 02	6 months	23 185	55.1	7.23	0.87	0.99	0.99	0.98
Sample 03	6 months	19 864	67.1	7.91	0.83	0.99	0.99	0.98
Sample 04	6 months	26 490	49.6	8.18	1.08	0.99	0.99	0.98
Sample 05	6 months	25 313	43.5	7.41	0.87	0.99	0.99	0.98
Sample 06	6 months	14 762	80.3	7.67	0.86	0.99	0.98	0.99
Sample 07	6 months	27 264	48.9	8.19	1.01	0.98	0.98	0.98
Sample 08	6 months	4500	97.7	8.10	0.67	0.99	0.99	0.99
Sample 09	6 months	25 067	42.6	6.80	0.81	0.99	0.98	0.98
Sample 10	6 months	26 311	42.8	7.36	0.96	0.99	0.99	0.98
Sample 11	6 months	20 781	48.7	5.80	0.83	0.99	0.99	0.98
Sample 12	3 months	23 180	41.4	6.83	0.93	0.99	0.99	0.98
Sample 13	3 months	26 284	44.8	7.38	0.95	0.99	0.99	0.98
Sample 14	3 months	24 689	48.1	6.94	0.84	0.99	0.99	0.97
Sample 15	3 months	26 586	48.3	6.64	0.97	0.98	0.99	0.98
Sample 16	3 months	24 695	40.7	5.82	0.82	0.99	0.98	0.98
Sample 17	3 months	18 194	69.9	6.01	0.67	0.99	0.99	0.97
Sample 18	3 months	23 009	51.9	6.87	0.99	0.99	0.99	0.97
Sample 19	3 months	17 837	75.5	7.88	0.85	0.99	0.99	0.98
*P. albiflos*	–	19 162	27.7	10.56	1.23	0.99	0.99	–
*P. staminae*	–	19 752	29	12.23	1.63	0.99	0.99	–

### Inferred relationships among individuals

We recovered 65 different plastid protein-coding sequences from the transcriptomes, 53 of which were present in a sufficient number of taxa to infer relationships (four samples) (Supplementary Data Table S3). The transcriptome data provided 63 different mitochondrial protein-coding sequences (genes or open reading frames), 55 of which were present in at least four samples (Supplementary Data Table S4).

The majority of relationships were poorly supported (UFboot ≤ 95 %) for both organelle phylogenies. The individuals did not form clades corresponding to ‘species’ based on taxonomic determinations (Supplementary Data Figs S2 and S3). Most relationships in the mitochondrial tree conflicted with relationships in the plastid tree (Supplementary Data Figs S2 and S3). In the mitochondrial tree, the outgroups, *P. staminae* and *P. albiflos*, were not monophyletic. The mitochondrial tree length (i.e. the sum of all branch lengths) was 0.286 subs/site, in comparison to the plastid tree length of 0.751 subs/site. The total parsimony-informative characters for the plastid dataset was 2698 sites, in comparison to 1060 sites for the mitochondrial dataset (Supplementary Data Fig. S4).

We recovered 11 683 nuclear orthologues shared by ≥10 of the 21 samples (19 ingroups and 2 outgroups). The relationships in the nuclear tree did not correspond to species identifications from either the taxonomist’s determinations or species assignments based on geolocation data, nor were they concordant with either organelle tree. However, unlike the organelle trees, the nuclear relationships were well supported, with nearly all relationships containing local posterior probabilities (≥0.95). The structure of the nuclear phylogeny was largely concordant with the relationships expected based on the ecology and geography of the island (Fig. 2).

### Variant calling and principal components analysis

FreeBayes called 492 539 SNPs across the 11 683 nuclear loci (orthologues used in the phylotranscriptomic analysis). After the data were filtered using the criteria outlined in the Materials and Methods, 10 218 high-quality SNPs across 2933 loci remained. The fraction of heterozygotes across the alleles followed a distribution similar to what would be expected under Hardy–Weinberg equilibrium, providing further evidence that this dataset is suitable for comparing gene flow among individuals (Supplementary Data Fig. S5). To account for linkage disequilibrium and avoid SNP-dense loci being more influential, we randomly retained one SNP per locus and ensured that the SNP was informative (i.e. variable across samples). The final result was 1900 informative SNPs after all filtering steps.

In our PCA, principal component (PC)1 and PC2 explained 13.98 and 10.38 % of the variation, respectively (Fig. 3). The samples did not form tight clusters in the PCA; however, the samples collected from the north-east (NE) side of the island aligned on the PC1 axis but not the PC2 axis, and vice versa for the south-west (SW) side. Samples 13 and 02, collected from the centre of the island, fell in the centre of the graph.

### Analysis of genetic diversity across the Dominican Republic

The FEEMS visualization showed a barrier to gene flow between the samples found on the NE and the SW side of the Cordillera Central mountain (Fig. 1B). The NE/SW barrier was corroborated by the conStruct analysis, which predicted a unique layer on each side of the mountain (Fig. 1). Samples 02 and 13 were collected between the NE and SW sides and were inferred to have a mixture of the NE and SW layers based on the conStruct analysis, with a greater proportion from the SW layer.

Samples 03, 04 and 05, collected on the eastern side of the Sierra de Bahoruco mountain range, were the geographically and genetically closest individuals on the SW side of the Cordillera Central and showed decreased gene flow with Sample 06, collected from the opposite side of the Sierra de Bahoruco. The FEEMS results did not suggest any decrease in gene flow by distance between Sample 13 and Samples 06, 07, 08 and 09, but they did show decreased gene flow by distance from Sample 02 to the other SW samples.

On the NE side, FEEMS showed gene flow between Samples 01, 10 and 12 in the Cordillera Central and between Samples 14, 15 and 17 in the Samaná Peninsula and Bay, but decreased gene flow between those and Samples 11, 16, 18 and 19. Gene flow also appeared to be reduced between the cluster of Samples 11 and 16 and the cluster of Samples 18 and 19, although the individuals in both clusters showed higher genetic similarities between each other than with any other samples.

## DISCUSSION

### RNA can be obtained from silica-preserved tissue

This study demonstrates that RNA can successfully be extracted and sequenced if the plant samples are stored in silica gel at room temperature. The plant tissue was never frozen after collection, differentiating this study from previous work extracting RNA from silica-dried samples (He *et al.*, 2022), which showed that storage of silica-dried plant tissue at −20 °C is sufficient for RNA extractions. The RIN values we obtained were generally lower than those recovered by He *et al.* (2022), and the RIN value of 2.3 for Sample 01 is considered low quality. However, the assembled transcriptome for Sample 01 was of similar quality to those of samples with superior RIN values (Table 1; Supplementary Data Table S2).

Historically, the stability of RNA before extraction has been a concern for transcriptome sequencing. However, we found that RNA in dried leaf material remained stable enough for short-read sequencing for ≥6 months. This suggests that transcriptomes might be obtained from plant tissues anywhere field collection is possible. The misconceptions about RNA stability might arise from differential gene expression studies, where there is a need to stop cellular processes quickly, or the desire to retain long sequences for PCR (Natarajan *et al.*, 2000). These misconceptions are likely to be reinforced by protocols requiring that tissue is frozen or stored in an RNA preservation solution (Logeman *et al.*, 1987; Yang *et al.*, 2017; Vennapusa *et al.*, 2020). Future work has the potential to provide valuable insight into the length of time that RNA remains intact and capable of providing transcriptomes, and to uncover any phylogenetic patterns in the rate of RNA degradation. As future work continues in silica-preserved tissue RNA extraction, it will be valuable to develop further protocols that maximize the efficiency of RNA extraction and ensure the accuracy of the sequenced sample.

### Reliable transcriptomes can be obtained from silica-preserved tissue

The field of historical RNA is in its infancy. The recent sequencing of the Tasmanian tiger transcriptome has demonstrated that expression profiles can be obtained from museum collections (Mármol-Sánchez *et al.*, 2023). Historical RNA provides a new source of gene expression data, provides new data for microRNA prediction and enables superior genome annotation of rare or extinct species.

Few studies have sought to obtain RNA from unpreserved tissue in the botanical world, limiting sources to silica-dried samples stored at −20 °C (He *et al.*, 2022) or seeds (Fordyce *et al.*, 2013). Seeds have been germinated after thousands of years (Sallon *et al.*, 2008), demonstrating that they provide natural protection against RNA degradation. Extraction of RNA from silica-preserved leaf tissue simplifies the transportation and shipping of tissue for RNA extraction. Although silica-preserved 6-month-old tissue is unlikely to qualify as historical, it provides promise that RNA is more stable than previously thought. To assess the quality of the RNA, we examined several metrics.

We tested whether the samples clustered based on the collection date, something that might occur owing to batch effects. In the most extreme example, the two most genetically and geographically similar individuals, Sample 11 and Sample 16, did not cluster based on collection date (Table 2). We examined the number of phylogenetic branches removed from each sample during the homologous gene tree cleaning. In this procedure, the sequences associated with the longest branches are removed from downstream analyses, because errors in homologue predictions can lead to long branches (Yang and Smith, 2014). However, museum degradation of DNA can also lead to long branches in phylogenies (McCormack *et al.*, 2016). Therefore, we expect highly degraded samples to be removed disproportionately from the homologous gene trees owing to long branches. We found no evidence that any sample had a disproportionately high removal rate, and the samples that lost the most branches were the outgroups (Table 2), both of which were preserved in liquid nitrogen (Palma‐Silva *et al.*, 2016).

We examined two metrics to determine the gene coverage: (1) how many genes within the samples are ≥70 % similar to the closely related pineapple genome; and (2) how many universal single-copy orthologues each sample had. For 15 of the 19 silica-preserved samples, more genes had ≥70 % sequence similarity than the outgroups. In contrast, the outgroups had the most universal single-copy orthologues (Table 2). The BUSCO analysis of He *et al.* (2022) showed similar results between the liquid nitrogen and the silica-dried sampling, indicating that the difference might be analytical; however, owing to the different sequencing runs it is difficult to determine the exact reason for this discrepancy.

To assess the quality of the transcriptomes, we used the program Transrate (Smith-Unna *et al.*, 2016) to calculate the trustworthiness of a contig based on the alignment edit distance, the *s*(*C*_nuc_) value. This value ranges between zero and one and is based on the number of reads that map to a given transcriptome and applies a penalty based on how often a mapped read contains an SNP. Therefore, a small penalty is introduced when a contig is from a heterozygous sample, because the alleles will cause disagreement for the position. Therefore, a good value will be close to one but unlikely to be 1.0. Of all the transcriptome samples, the lowest average value across the nuclear data was >0.98, indicating a high level of agreement between the reads and the assembled contigs.

### *Morphology and collection location provide conflicting species delimitation for* Pitcairnia

There are six named species of *Pitcairnia* endemic to the Dominican Republic. Of these, five species have been described formally (Acevedo-Rodríguez and Strong, 2012): *P. domingensis*, *P. elizabethae*, *P. fuertesii*, *P. jimenezii* and *P. samuelsonii.* The sixth, *P. ariza-juliae*, was identified as new from herbarium specimens by Robert W. Read from the Smithsonian Institution (JBSD: Liogier 27724; Zanoni 15005; Zanoni 22952) but never described formally. Although this is not a published name, the specimens listed above are identified as such in the herbarium specimens. The species in Ayiti were described by Carl Mez (*P. fuertesii*) and Lyman B. Smith (all others) between 1913 and 1964 from pressed specimens (Smith and Downs, 1974).

The Flora Neotropica monograph of the Pitcairnioideae subfamily (Smith and Downs, 1974) remains the most comprehensive work published on the group. However, the monograph does not address how the taxa are different from each other, and the identification key (which covers all species of *Pitcairnia*) does not group the five described species together in the same subkeys. *Pitcairnia elizabethae* is characterized as the shortest, reaching a maximum height of 28 cm, and *P. fuertesii* and *P. jimenezii* are the tallest, both reaching heights of 100 cm. *Pitcairnia domingensis* and *P. jimenezii* are glabrous, whereas the rest are densely covered in trichomes on the abaxial side of the leaves. *Pitcairnia jimenezii* has no marginal spines, whereas the rest have spines of varying length and density. The monograph does not describe any diagnostic characters unique to individual species. From our field observations, the most pronounced morphological disparities are in the length and width of leaves, the length of the flower stalk, the density of trichomes covering the leaves, and the density and size of marginal spines (Supplementary Data Figs S6 and S7).

The distribution of each species was thoroughly documented and outlined by Smith and Downs (1974). *Pitcairnia fuertesii* is the only species widely distributed across Ayiti, with specimens from the Samaná Peninsula, Sierra de Bahoruco and the Cordillera Central mountain ranges in the Dominican Republic and from the Massif du Nord and Massif de la Selle mountain ranges in Haiti. *Pitcairnia domingensis* is circumscribed to Samaná, *P. elizabethae* from Massif du Nord in Haiti and Sierra de Bahoruco in the Dominican Republic, *P. jimenezii* from Puerto Plata Province, and *P. samuelsonii* from the Massif du Nord and Massif de la Selle in Haiti and the San Juan de la Maguana province in the Dominican Republic. Since then, new records for each species have been recorded in museum specimens.

The lack of an identification key specific to the species from Ayiti or the Caribbean has made identification of *Pitcairnia* specimens difficult. To account for this and maximize the diversity of collections, sampling in this study covered the historically reported range of the genus across the Dominican Republic. Some locations in the centre of the Cordillera Central have been altered by human development, and *Pitcairnia* was not found during fieldwork.

### Phylogenetic analyses of the nuclear and organellar genomes do not support species delimitations

The two methods of species identification (taxonomist and geolocation) disagreed for 12 of the 17 samples, highlighting the need to examine the relationships further. None of the measurements performed on the samples (trichome density, size of marginal spines, and leaf length and width) corroborate either classification scheme (Supplementary Data Fig. S6). Given that *Pitcairnia* species are delimited based on quantitative traits, these differences might be the result of plasticity, which might be exacerbated by the diverse ecology of the Dominican Republic. Molecular phylogenies can often help with species delimitation, because molecular data provide a large number of characters with which to estimate relationships.

The plastome and mitochondrial genomes remain powerful tools for molecular phylogenetics; they are two of the most character-rich markers, are often considered each to have a single evolutionary history and are obtainable from transcriptome data (Morales-Briones *et al.*, 2021). Based on a four-plastome gene molecular phylogeny, the *Pitcairnia* of the Dominican Republic were inferred to be monophyletic (Schubert, 2017). In our dataset, each organelle contained >50 phylogenetically informative protein-coding sequences. However, based on the low support for relationships, the organellar genomes proved insufficient for reliable estimation of species relationships. Mitochondrial and plastid trees often have concordant topologies (Tyszka *et al.*, 2023), but in our case, the lack of resolution and conflict between the two provide additional evidence that the relationships inferred by the organelle trees should not be trusted. Further examination of the amount of molecular evolution between samples shows that the topologies are inferred from limited information, as reflected by the small total tree length (Supplementary Data Figs S2 and S3) and low level of parsimony-informative characters (Supplementary Data Fig. S4).

We also examined nuclear genome-based relationships using transcriptome data. One of the difficulties for inferring relationships using nuclear data is phylogenetic conflict, specifically incomplete lineage sorting. Given that *Pitcairnia* appears to have experienced a rapid radiation on the island of Ayiti, one might expect there to be high levels of incomplete lineage sorting. To accommodate incomplete lineage sorting, we used the coalescent-based maximum quartet support species tree method. This approach resulted in a species tree where almost all relationships were well supported (Fig. 2). Despite the strong support, the individuals did not form clades with their respective species, hence neither classification method was validated. Instead of clustering based on taxonomic species determinations, the individuals clustered based on geography and ecology. Specifically, a major split in the tree was found between samples on the NE and the SW side of the Cordillera Central.

### *Mountain ranges in the Dominican Republic are barriers to gene flow among* Pitcairnia *individuals*

Based upon the short molecular branch lengths of the inferred phylogenies, indicating minimal molecular evolution among individuals, the data appeared suitable for population genetic analyses. Previous work has shown that transcriptomes can provide a data-rich source to infer patterns of gene flow (Raduski *et al.*, 2021). Therefore, we proceeded with a series of population genetic analyses to investigate the relationships among the individuals further. In the PCA, the individuals did not form clear clusters with respect to the first two PC axes (Fig. 3), indicating that there were not clearly differentiated groups that could be binned into separate populations for downstream analyses.

The program conStruct (Bradburd *et al.*, 2018) was used to infer gene flow across the island. ConStruct assumes several distinct ancestral populations called layers. Each individual is inferred to have some proportion of ancestry from each layer, and the more similar the individuals, the more similar the composition of layers will be. Unlike other approaches to analysis of population structure, conStruct incorporates spatial information into the analysis. A complementary approach to analysing and visualizing isolation by distance was conducted using FEEMS, which complements conStruct because it is robust to sparse sampling and functions well under simulated coalescent processes (Marcus *et al.*, 2021). FEEMS determines whether the amount of genetic distance between individuals is what would be expected based on geographical distance. Any deviations from that might indicate a barrier to gene flow.

The split in the phylogeny corresponding to whether samples were collected NE or SW of the Cordillera Central was also reflected in the population genetic analyses (the PCA, conStruct and FEEMS). The Cordillera Central is the tallest mountain range in the Caribbean and is a well-documented barrier to gene flow in some palms (Rodríguez-Peña *et al.*, 2014) and a predicted driver of speciation in *Podocarpus* (Nieto‐Blázquez *et al.*, 2021). Based on all analyses, the Cordillera Central appears to be the best explanation for the genetic differentiation between the SW and the NE *Pitcairnia* individuals. All analyses performed resulted in concordant results, indicating robust support for this result regardless of the approach used.

On the NE side of the Cordillera Central, the relationship between species largely matches geographical distances; for example, Samples 18 and 19 and Samples 11 and 16 cluster together in both the PCA and the phylogeny. A comparison of Samples 11 and 16, in particular, illustrates how phenotypic plasticity might be responsible for the morphological differences observed between individuals (Supplementary Data Fig. S8). These two samples are the least genetically differentiated of any pair of samples and were collected 1.31 linear km apart, but they show greater differences in the quantitative morphological traits. The leaves of Sample 11 reached 140 cm, were softer and appeared greener in colour because of a reduced density of trichomes. When these plants were collected, they were growing on the side of the road, and although close to and facing the ocean, they were found under lush vegetation. Sample 16 was shorter, with coarse, greyish leaves reaching 35 cm, and was growing on the cliffs directly facing the ocean.

A similar pattern of isolation by distance was observed for samples from the SW side of the island. However, despite Sample 06 being geographically closer to Samples 03, 04 and 05, it was genetically closer to Samples 07, 08 and 09. The shared ecology might explain the genetic similarity, because Samples 06, 07, 08 and 09 were from drier habitats on the southern side of the Sierra Neiba mountain range, in comparison to the wetter habitats of Samples 03, 04 and 05. A pattern of decreased gene flow owing to the Sierra de Neiba and Sierra de Bahoruco has been documented previously in the small mammal *Solenodon* (Turvey *et al.*, 2016) and the cactus *Leptocereus* (Majure *et al.*, 2021).

Hummingbirds have been identified as the main pollinators of *Pitcairnia* (Benzing, 2000). Fumero-Cabán and Meléndez (2007) corroborated this for *Pitcairnia angustifolia* in Puerto Rico, identifying *Anthracothorax viridis*, the long-billed hummingbird, as the main pollinator. Although there are no studies or reported observations for the reproductive biology of *Pitcairnia* in Ayiti, similarities in floral morphology and habitats allow for the reasonable assumption that *Anthracothorax dominicus* would fulfil that role in the Dominican Republic. *Anthracothorax dominicus* is present throughout the island (GBIF.org., 2023), which does not suggest that pollinator specificity would drive differences between the populations.

### Conclusions

This study provides several advances for transcriptomics in evolutionary biology. We found that RNA for short-read sequencing can be extracted from leaf samples dried in silica gel and stored at room temperature for ≤6 months. Using this transcriptome sequence data, we demonstrate that the *Pitcairnia* of the Dominican Republic does not show the predicted genetic isolation expected for multiple species, and the Cordillera Central is the main isolator to gene flow. These results demonstrate promise for obtaining transcriptomes from any plant growing where tissue collection is possible.

## SUPPLEMENTARY DATA

Supplementary data are available at *Annals of Botany* online and consist of the following.

Figure S1: RNA extraction protocol using Sigma Spectrum™ Plant Total RNA Kit. Figure S2: inferred relationships among individuals using plastome data. Figure S3: inferred relationships among individuals using mitochondrial data. Figure S4: parsimony-informative characters across the protein-coding sequences for each dataset. Figure S5: the distribution of allele frequency for the nuclear data compared with that expected based on Hardy–Weinberg equilibrium. Figure S6: morphological differences among the named species of *Pitcairnia*. Figure S7: examples of variation of two morphological characters measured. Figure S8: although closely related, Samples 11 (left) and 16 (right) show significant variation in size, trichome density and leaf coarseness. Table S1: sample numbers, collections and their respective herbarium numbers at the National Herbarium of the Dominican Republic (JBSD). Table S2: full summary of sequenced samples and the resulting transcriptomes produced. Table S3: plastome genes recovered. Table S4: mitochondrial protein-coding sequences recovered.

mcae002_suppl_Supplementary_Figures_S1-S8_Tables_S1-S4
