# Correction to: Transcriptome data from silica-preserved leaf tissue reveal gene flow patterns in a Caribbean bromeliad

**DOI:** 10.1093/aob/mcae052

**Published:** 2024-04-03

This is a correction to: Natalia Ruiz-Vargas, Karolis Ramanauskas, Alexa S Tyszka, Eric C Bretz, May T S Yeo, Roberta J Mason-Gamer, Joseph F Walker, Transcriptome data from silica-preserved leaf tissue reveal gene flow patterns in a Caribbean bromeliad, *Annals of Botany*, 2024; mcae002, https://doi.org/10.1093/aob/mcae002

In the originally published version of this paper, the map in Figure 1b showed the island’s elevation instead of the FEEMS results. Figure 1a remains the same.

**Figure 1:**
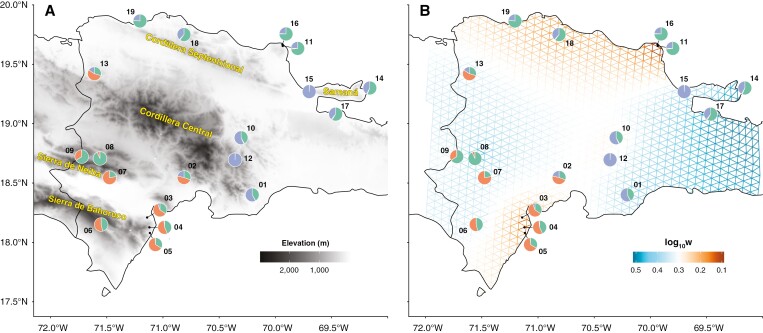
(Corrected)

This error has been corrected in the paper.

